# Cannabinoid Receptor 2-63 QQ Variant Is Associated with Persistently Normal Aminotransferase Serum Levels in Chronic Hepatitis C

**DOI:** 10.1371/journal.pone.0099450

**Published:** 2014-06-18

**Authors:** Nicola Coppola, Rosa Zampino, Caterina Sagnelli, Giulia Bellini, Aldo Marrone, Maria Stanzione, Nicolina Capoluongo, Adriana Boemio, Carmine Minichini, Luigi Elio Adinolfi, Sabatino Maione, Emanuele Miraglia Del Giudice, Evangelista Sagnelli, Francesca Rossi

**Affiliations:** 1 Department of Mental Health and Public Medicine, Second University of Naples, Naples, Italy; 2 Internal Medicine and Hepatology, Second University of Naples, Naples, Italy; 3 Department of Clinical and Experimental Medicine and Surgery, Second University of Naples, Naples, Italy; 4 Department of Experimental Medicine, Second University of Naples, Naples, Italy; 5 Department of Pediatrics, Second University of Naples, Naples, Italy; Centers for Disease Control and Prevention, United States of America

## Abstract

**Background and Aim:**

To evaluate in anti-HCV-positive patients the clinical impact of the rs35761398 variant of the *CNR2* gene leading to the substitution of Gln (Q) of codon 63 of the cannabinoid receptor 2 (CB2) with Arg (R).

**Patients and Methods:**

253 consecutive anti-HCV-/HCV-RNA-positive patients were enrolled, of whom 53 were HCV carriers with persistently normal ALT (PNALT group) and 200 had a history of steadily abnormal serum ALT values (abnormal ALT group). All patients were naive for antiviral therapy and were screened for the CNR2 rs35761398 polymorphism by a TaqMan assay.

**Results:**

Subjects in the PNALT group, compared with those in the abnormal ALT group were older (58.5±12 vs. 50.7±12.4 years, p = 0.001), more frequently female (66% vs. 42%, p = 0.003), with lower body massindex (BMI) (24.5±3.1 vs. 26.6±4.6, p = 0.003), and more frequently with HCV genotype 2 (43.1% vs 17.7%, p = 0.0002) and CB2-63 QQ variant (34% vs. 11%, p = 0.0001). Considering all 253 patients, no difference in the demographic, biochemical, or virological data was observed between patients in the different CB2-63 variants. The logistic regression analysis identified CB2-63 QQ, HCV genotype 2, older age and lower BMI as independent predictors of PNALT (p<0.00001).

**Discussion:**

The CB2-63 QQ variant in HCV patients was independently associated with the PNALT status.

## Introduction

The Cannabinoid (CB) receptors are seven membrane domain receptors of the G-coupled receptor superfamily that are activated by endogenous (endo) or exogenous (phyto and synthetic) cannabinoids [1], [2]. Among the endocannabinoids, anandamide (AEA) was the first discovered, followed by 2-arachidonoylglycerol (2-AG) [1], [2]. Two types of CB receptors are known, type 1 (CB1) and type 2 (CB2): CB1 is predominantly expressed in the central nervous system but also in the lung, liver and kidney, and CB2 in the immune and immune-derived cells (lymphocyte T and B cells, etc.) [3], [4] .CB2 is also highly expressed in Kupffer cells, resident macrophages in the liver, which, due to their phagocytic activity, play an essential role in the acute and chronic responses of the liver to toxic and infectious agents [5].

A polymorphism at codon 63 of the Cannabinoid Receptor 2 gene (CNR2) leads to the substitution of glutamine, Gln (Q), with arginine, Arg (R), causing a different polarization state of the protein; the CB2 variants have been demonstrated to affect differently the ability of the CB2 receptor to exert its inhibitory function[5], [6]. Specifically, *in-vitro* T lymphocytes from CB2-63 RR homozygotes showed an approximately two-fold reduction in the endocannabinoid-induced inhibition of proliferation compared to cells from CB2-63 QQ homozygotes [6], [7].

The hepatitis C virus (HCV) infects over 170 million people worldwide and is a leading cause of cirrhosis and hepatocellular carcinoma [8]. The severity of chronic hepatitis C (CHC) is highly variable among patients and over time. Some individuals show a mild indolent clinical course for decades, others rapidly progress to end-stage liver disease [9], whereas about 20–30% of cases remain asymptomatic with normal serum alanine aminotransferase (ALT) levels lifelong. According to the Italian guidelines, the definition of an anti-HCV carrier with persistently normal ALT (PNALT) serum values applies to a subject with at least 9 consecutive ALT normal values observed at two-monthly intervals over an 18-month observation period[10]and identifies a subclinical form of CHC. The majority of carriers with PNALT show histological evidence of necroinflammation and fibrosis, but the liver lesions are clearly less severe than those observed in subjects with abnormal ALT [11], [12]. Most of them show a slow fibrosis progression [13], but liver cirrhosis may develop after an ALT flare-up has occurred [14].

Several factors have been found to be linked to the progression and severity of CHC, including virus-related (duration of infection) and host factors (interleukin-28B polymorphism), co-morbidities (coinfection with human immunodeficiency virus, HIV, or hepatitis B virus, HBV, insulin-resistance, high body mass index, immunosuppression), and lifestyle factors (alcohol intake, cannabis use) [15]–[20]. Moreover, the data from a recent study suggested the involvement in CHC of the rs35761398 variant of the CNR2 gene encoding for CB2, since the CB2-63 QQ variant was associated with more severe necroinflammation in anti-HCV-positive patients with abnormal ALT [21]. Instead, only a few published data on factors possibly associated with the subclinical form of chronic HCV infection are available at present.

The present paper analyzes the role of CB2 variants in 53 consecutive HCV carriers with PNALT in comparison with 200 consecutive patients with abnormal ALT.

## Patients and Methods

### Patients

Two Liver Units in the Campania region (southern Italy) participated in the study. These two centers have cooperated in several clinical investigations using the same clinical approach and the same laboratory methods [21]–[23].

Two hundred and fifty-three consecutive Italian patients with chronic HCV infection (anti-HCV- and HCV-RNA-positive) for 18–36 months were included in the study. The patients were first observed from July 2009 to December 2012 and were naive for antiviral therapy at that time. Of the 253 patients enrolled, 53 (20.9%) were HCV carriers with PNALT (PNALT group) and 200 (79.1%) had a history of steadily abnormal serum ALT values (abnormal ALT group).

All 253 cases underwent complete physical examination, liver ultrasound scan, liver function tests, assessment of triglycerides, cholesterol, blood cell counts, alpha-fetoprotein and HBV, HCV, hepatitis delta virus (HDV) and HIV serum markers. All patients enrolled were asymptomatic, negative for anti-HIV, HBsAg and anti-HDV. On a specific home-made questionnaire none stated ongoing intravenous drug addiction at admission. Alcohol abuse was defined as the consumption of alcohol exceeding 30 g per day for females and 40 g per day for males in the last 6 months; the patient’s statements regarding intravenous drug addiction and alcohol abuse were corroborated by the patient’s family and by serum/urine tests in uncertain cases.

Liver biopsy was performed for 186 (73.5%) patients. The liver biopsy was proposed for all patients in the abnormal ALT group, but it was not performed for 29 (11.5%) because of refusal in 20 cases and contraindication in 9. In the PNALT group the liver biopsy was advised for the 18 patients aged 50 to 65 years with genotype 1 and was performed only for 15 of these because of refusal by the remaining 3 patients. Liver specimens were fixed in formalin, embedded in paraffin and stained with hematoxylin-eosin and Masson’s trichrome stain. In each case, the liver specimens were more than 2 cm in length and had more than 11 portal tracts. Liver biopsies were examined by a pathologist who, unaware of the clinical and laboratory data, used the Ishak’s scoring system to grade necroinflammation and fibrosis [24]. To assess liver steatosis we used a home-made scoring system obtained with a partial modification of Kleiner’s scoring system for non-alcoholic fatty liver diseases [25], extensively reported in previously published papers [12], [21], [23]. Briefly, score 1 identifies the presence of fatty deposition in 1–10% of hepatocytes, score 2 in 11–31%, score 3 in 31–60% and score 4 in >60%. Samples of serum and whole blood were obtained for each patient at the time of enrolment and stored at −80°C until used for this investigation.

As a control group, six hundred normal subjects were evaluated for the CB2-63 polymorphism and ALT serum value.

### Ethics Statement

All procedures used were in accordance with the international guidelines and with the Helsinki Declaration of 1975 and revised in 1983. The study was approved by the Ethics Committee of the Azienda Ospedaliera Universitaria of the Second University of Naples. All patients provided written informed consent for the collection and storage of biological samples and for the anonymous use of their data in clinical research.

### Routine Analysis

HBV and HDV serum markers were sought using commercial immunoenzymatic assays (Abbott Laboratories, North Chicago, IL, USA, for HBsAg, anti-HBs and anti-HBc, and DiaSorin, Saluggia, VC, Italy, for anti-HDV). The anti-HCV antibody was sought using a 3rd generation commercial immunoenzymatic assay (Ortho Diagnostic Systems, Neckargemund, Germany). Antibodies to HIV 1 and 2 were sought using a commercial ELISA (Abbott Lab., North Chicago, Ill, USA). Liver function tests (ALT; aspartate aminotransferase; γ-glutamyltransferase, etc.) were performed by the routine serological methodsin Cobas Modular 6,000 automated analyzer using c501 biochemistry modules (Roche Diagnostics Ltd, Rotkreuz, Switzerland).

### HCV Genotype and Viral Load

HCV genotype was determined using HCV genotype assay Lipa (Bayer, France), following the manufacturer’s instructions.

Viral RNA was extracted from 140 µl of plasma samples using a microspin column (QIAamp RNA viral kit, Qiagen GmbH, Hilden, Germany). The HCV RNA was quantified by performing a real-time polymerase chain reaction (PCR) in a Light cycler 1.5 (Roche Diagnostics, Branchburg, NJ, USA), as reported in a previous paper [26]; by this method, the detection limit in plasma samples is estimated at around 40 IU/mL.

### Genetic Analysis

Genomic DNA was extracted from peripheral whole blood with a DNA extraction kit (Roche Diagnostics, Branchburg, NJ, USA) after written informed consent. Molecular screening for the CNR2 rs35761398 polymorphism (CAA/CGG) underlying the CB2 Q63R substitution was performed using a TaqMan Assay (Real Master Mix Probe, 5 PRIME, Germany). Primers and probes were the following: sense primer 5′-GTGCTCTATCTGATCCTGTC-3′ and anti-sense primer 5′-TAGTCACGCTGCCAATC-3′; AA-probe 5′-CCCACCAACTCCGC-3′ and GG-probe 5′-CCCACCGGCTCCG-3′ (PRIMM, Milan, Italy). Both PCR and post-PCR allelic discrimination were carried out on an ABI PRISM 9600 apparatus (Applied Biosystems, Foster City, CA). Random samples were confirmed by direct PCR sequencing consisting of 94°C for 4 min followed by 31 cycles of 94°C for 30 s, 60°C for 30 s and 72°C for 30 s with forward 5′-GAGTGGTCCCCAGAAGACAG-3′ and reverse 5′-CACAGAGGCTGTGAAGGTCA-3′ primers. PCR products were analyzed using an ABI PRISM 3100 automated sequencer (Applied Biosystems, Foster City, CA) and the Big Dye Terminator reaction kit (Applera, Foster City, CA), according to the manufacturer’s instructions. All primers were chosen using Primer3 software (http://primer3.sourceforge.net/).

DNAs were tested also for the rs12979860 polymorphism of the IL-28B genotype (Roche Diagnostics, Branchburg, NJ, USA).

### Statistical Analysis

Continuous variables were summarized as mean and standard deviation, and categorical variables as absolute and relative frequencies. Differences in the mean values were evaluated by an unpaired Student t-test, and the chi-squared test was applied to categorical variables. Not normally distributed variables (AST, ALT, HCV viral load) were log-transformed before the analysis, and the mean values are shown in Tables 1 and 2. A p value<0.05 was considered to be statistically significant. All independent variablesat univariate analysis with a biological plausibility to the aim of the study were included in the multivariate analysis using a general linear model. The statistical analysis was performed using Statgraphics Centurion XV.II (Adalta, Arezzo, Italy; Statpoint Technologies Inc., Virginia, USA).

**Table 1:** Demographic, genetic, virological, biochemical and histological data according to clinical condition.

	PNALT group	Abnormal ALT group	P-value
**No. of patients**	53	200	
**Males, no. (%)**	18 (33.9)	116 (58)	0.003
**Age, years (M ± SD)**	58.50±12.04	50.70±12.38	0.0001
**BMI, (M** ± **SD)**	24.51±3.14	26.56±4.63	0.003
**AST, × n.v. (M ± SD)**	0.58±0.27	1.84±1.36	0.0001
**ALT, × n.v. (M ± SD)**	0.58±0.29	2.38±1.62	0.0001
**gGT, IU/ml (M ± SD)**	39±54	53±45	0.055
**Glucose, mg/dl (M ± SD)**	102±33	95.37±21,72	0.067
**Iron, ug/dl (M ± SD)**	106±32	112±50	0.408
**Ferritin, ng/ml (M ± SD)**	132±106	157±127	0.189
**Transferrin, (M ± SD)**	290±45	287±46	0.552
**Triglycerides, mg/dl (M ± SD)**	96±49	106±58	0.251
**Total cholesterol, mg/dl, (M** ± **SD)**	173±23	177±42	0.506
**HCV RNA, IU×10^3^ (M ± SD)**	3,559±5,087	2,367±5,746	0.171
**HCV genotype, no. (%):**			
** 1**	28 (54.9)	131 (68.2)	2 vs non-2: 0.0002
** 2**	22 (43.1)	34 (17.7)	
** 3**	1 (1.96)	21 (10.9)	
** 4**	0	4 (2.1)	
** Missing**	2	0	
**IL28-B, no. (%):**			
** CC**	20 (37.7)	53 (26.5)	CC vs. non CC: 0.151
** CT**	27 (50.1)	121 (60.5)	
** TT**	6 (11.3)	26 (13)	
**CB2**-**63, no. (%):**			
** QQ**	18 (34)	22 (11)	QQ vs non-QQ: 0.0001
** QR**	19 (35.8)	110 (55)	
** RR**	16 (30.2)	68 (34)	
**Patients with LB, no. (%):**	15 (28.3)	171 (88.5)	////
**HAI score, (M ± SD)**	3.47±2.47	6.04±3.50	0.0001
**Fibrosis score, (M ± SD)**	1.27±0.59	2.39±1.44	0.0001
**Steatosis score, (M ± SD)**	0.33±0.62	1.21±1.26	0.0001

M: mean; SD: standard deviation; BMI*:* body mass index; AST: aspartate-aminotransferase; x n.v.: times normal value; ALT: alanine aminotransferase; gGT: g-glutamyltransferase; LB: liver biopsy.

**Table 2:** Demographic, biochemical and histological characteristics in HCV patients according to CB2 63 genotype variants.

	CB2 QQ	CB2 QR	CB2 RR
**No. of patients**	40	129	84
**Males, no. (%)**	19 (47.5)	68 (52.7)	47 (56)
**Age, years (M ± SD)**	55.4±12.2	51.6±12.1	51.9±13.6
**BMI (M ± SD)**	24.97±5.77	26.17±4.01	26.30+4.03
**AST × n.v. (M ± SD)**	1.61±1.54	1.56±1.22	1.58+1.37
**ALT × n.v. (M ± SD)**	1.76±1.85	1.71±2.12	1.95±1.34
**Total cholesterol, mg/dl (M ± SD)**	163±31	178+43	178±36
**Triglycerides, mg/dl (M ± SD)**	106±50	104+62	107±49
**Iron, ug/dl (M ± SD)**	138±51	108±43	111±58
**Ferritin, ng/ml, (M ± SD)**	160±72	154±126	129±123
**Transferrin, (M ± SD)**	313±53	289+36	278±58
**gGT IU/ml, (M ± SD)**	62±66	51±48	49±32
**Glucose mg/dl, (M ± SD)**	90±11	97±25	96±21
**HCV RNA, IU×10^3,^(M ± SD)**	2,394±2,470	2,109±3,872	3,620±8,239
**HCV genotype, no. (%):**			
** 1**	27 (69.2)	83 (66.4)	50 (63.3)
** 2**	7 (17.9)	27 (21.6)	22 (27.8)
** 3**	3 (7.7)	14 (11.2)	5 (6.3)
** 4**	2 (5.1)	1 (0.8)	2 (2.5)
** Missing**	1	4	5
**Patients with LB, no. (%)**	21 (52.5)	107 (82.9)	58 (69.04)
**HAI score (M ± SD)**	7.81±4.08^a^	5.60±3.33^b^	5.55±3.47^c^
**Fibrosis score (M ± SD)**	2.67±1.77	2.24±1.39	2.28±1.35
**Steatosis score (M ± SD)**	1.19±1.25	1.17±1.19	1.22±1.23
**Patients with PNALT, no. (%)**	18 (45)^d^	19 (14.7)^e^	16 (19.04)^f^
**IL28-B, no. (%):**			
** CC**	11 (27.5)	41 (31.8)	21 (25)
** CT**	24 (60)	77 (56.7)	47 (56)
** TT**	5 (12.5)	11 (8.5)	16 (19.04)

M: mean; SD: standard deviation; BMI*:* body mass index; AST: aspartate-aminotransferase; x n.v.: times normal value; ALT: alanine aminotransferase; gGT: g-glutamyltransferase; LB: liver biopsy.

Differences significant to the statistical analysis: a vs. b: p = 0.008; a vs. c: p = 0.017; d vs. e: p = 0.0001; d vs. f: p = 0.005.

## Results

The differences in the initial characteristics between the 53 subjects with PNALT and the 200 with abnormal ALT are shown in Table 1. Subjects in the PNALT group, compared with those in the abnormal ALT group, were older [mean± standard deviation (SD) 58.5±12 vs. 50.7±12.4 years, p = 0.001], more frequently female (66% vs. 42%, p = 0.003), with lower body mass index (BMI) (24.5±3.1 vs. 26.6±4.6, p = 0.003), and more frequently with HCV genotype 2 (43.1% vs. 17.7%, p = 0.0002) and the CB2-63 QQ variant (34% vs. 11%, p = 0.0001). To confirm our CB2-63 data, we considered as two separate cohorts the two series of subjects with HCV chronic infection included at the two enrolling Liver Units and compared the results obtained. Similar prevalences of CB2-63 QQ variant were observed in these two cohorts, both in PNALT subjects (1^st^ cohort 31% of 35 subjects; 2^nd^ cohort 38% of 18 subjects) and chronic hepatitis patients (1^st^cohort 10.7% of 130 patients; 2^nd^ cohort 11.4% of 70 patients).

Also the IL28-B CC genotype was more frequently observed in the PNALT than in the abnormal ALT group (37.7% vs. 26.5%), a small difference not significant to the statistical analysis (p = 0.1). Considering only patients with liver biopsy, the 15 in the PNALT group showed a lower histological activity index (HAI) (3.47±2.47 vs. 6.04±3.5, p = 0.0001), lower fibrosis (1.27±0.59 vs. 2.39±1.44, p = 0.0001) and lower steatosis scores (0.33±0.62 vs. 1.21±1.26, p = 0.0001) than the 171 patients in the abnormal ALT group (Table 1). The 118 patients with a BMI over 25 had a higher steatosis score than the 68 with a lower BMI (1.5±1.4 vs. 0.8±1.05).

Considering all 253 patients with chronic HCV infection in the present study, the prevalence of PNALT subjects was significantly higher in the CB2-63 QQ than in the QR and RR subgroups (45% vs. 14.7%, p<0.0001, and 19.04%, p<0.005, respectively), whereas no difference in demographic, biochemical, or virological data was observed between patients in the different CB2-63 subgroups (Table 2). No association of the HCV load with the PNALT status or a CB2-63 variant was found in the present study (Tables 1 and 2), which is in agreement with previous investigations never displaying HCV load associated with clinical, biochemical and histological findings [27]–[29]. In addition, no significant difference in the ALT serum values was observed between subgroups established on the basis of the CB2 variants, most probably due to the fact that of the 253 subjects investigated, 200 were chronic hepatitis patients with increased ALT values and only 53 were PNALT subjects with normal ALT, too small a number to influence substantially the mean ALT levels in the three CB2 subgroups. Also in the 600 normal controls, no significant difference in the ALT values was observed in the three CB2-63 variants.

Of the 186 patients who underwent liver biopsy (171 in the abnormal ALT group and 15 in the PNALT group), the 21 with CB2-63 QQ showed a higher (mean±SD: 7.81±4.1) HAI than the 107 with CB2-63 QR (5.6±3.3, p = 0.008) and the 58 with CB2-63 RR (5.5±3.5, p = 0.017), whereas no statistically significant differences were observed in the degree of liver fibrosis and steatosis. This analysis was not performed separately for the abnormal ALT group and the PNALT group because the latter included only 15 patients with liver biopsy, of whom only 2 with the QQ and 3 with the QR variant.

The prevalence of subjects with PNALT was higher (45%) in the 40 with CB2-63 QQ than in the 129 with CB2-63 QR (14.7%, p<0.0001) and in the 84 with CB2-63 RR (19%, p = 0.005).

To confirm the association between the CB2-63 variants and the status of PNALT, a multivariate logistic regression analysis was performed (Table 3). The analysis included the CB2-63 QQ variant versus non-QQ, HCV genotype 2 versus non-2 and other potential confounding factors like age, sex and BMI. After all the confounding effects of the other risk factors were removed, the CB2-63 QQ variant, HCV genotype 2, older age and lower BMI were the only independent predictors of PNALT (p<0.00001).

**Table 3:** Logistic regression between CB2-63 variants and PNALT.

Analysis of Variance for PNALT					
Source	Sum of Squares	df	Mean Square	F-Ratio	*p*-Value
Model	10.59271648	7	1.51324521	10.18	**0.0000**
Residual	22.60103352	152	0.14869101		
Total (Corr.)	33.19375	159			
**Single factor contribution**					
**Source**	**Sum of Squares**	**df**	**Mean Square**	**F-Ratio**	***p*** **-Value**
**CB2**-**63 QQ vs non-QQ**	2.451458816	2	1.225729408	8.24	**0.0004**
**Age**	1.125386271	1	1.125386271	7.57	**0.0067**
**Sex**	0.066793766	1	0.066793766	0.45	0.5037
**BMI**	3.335028877	1	3.335028877	22.43	**0.0000**
**HCV genotype 2 vs non-2**	2.708676302	1	2.708676302	18.22	**0.0000**

## Discussion

This study analyzed the role of a functional polymorphism of the cannabinoid receptor type 2 in a cohort of 253 patients with HCV chronic infection and found the CB2-63 QQ variant independently associated with the PNALT status. Besides the CB2-63 QQ variant, the multivariate analysis identified another three factors independently associated with the PNALT status: HCV genotype 2, an older age and a lower BMI. HCV genotype 2 and a lower BMI have been suggested as independent predictors of PNALT in previous studies [10], [30], [31], and confirmed in the present investigation. Instead, this is the first time the CB2-63 QQ variant and an older age have been cited as independent predictors of the PNALT status.

The association between the CB2-63 QQ variant and the status of HCV carrier with PNALT may be the result of a prolonged strong inhibition of the T cells with QQ variants, with a consequently less vigorous immune response against infected hepatic cells. Support for this hypothesis comes from an *in-vitro* study on the CB2-mediated inhibition of T-cell proliferation, normal with T cells deriving from CB2-63 QQ subjects and reduced two-fold with T cells from subjects with the RR homozygous variant [7]. In fact, aggressive autoimmune pathologies such as celiac disease and childhood immune thrombocytopenic purpura have been found to be associated with the CB2-63 RR variant [32], [33].

It has been recently suggested that HCV-related proteins induce prolonged activation of liver Kupffer cells, leading to the accumulation of inflammatory cytokines that contributes to liver damage [34]. These cells, however, may also express a range of polarized phenotypes, including the M1proinflammatory phenotype and the M2 alternative phenotype involved in the resolution of inflammation and wound healing. It has beendemonstrated that CB2 stimulation inhibits pro-inflammatoryM1 polarization and favors the transition to the anti-inflammatory M2phenotype [35]–[37]. This transition might be more frequently expressed in CB2-63 QQ than in RR Kupffer cells due to the above-mentioned reduced inhibition of T-cell proliferation in subjects with CB2-63 RR. This transition may favor the progression from CHC to the PNALT status and explain, at least in part, the association observed between the QQ homozygous variant and the PNALT status.

The mean age of the PNALT subjects in the present study was 8 years older than that in the abnormal ALT group, suggesting that a substantial percentage of them may have reached the PNALT status through a variety of immunological conditions, from an active cellular immune response at the time of acute hepatitis C and in the initial stage of HCV carriage to a subsequent inhibition/failure of the cellular immune response favoring the progressionto the clinical and histological profile characteristic of PNALT. Such inhibition/failure of the immune response has been demonstrated to include type 1 helper T-cell (Th1) hypo-responsiveness, cytotoxic T lymphocyte (CTL) exhaustion, excessive function of regulatory T cells (Treg), and failure of lymphoid cells via direct binding and/or infection in B cells, T cells, natural killer cells and dendritic cells [38]–[43]. Since in CHC the liver damage is mainly induced by Th1- and/or CTL-related responses [44]–[46], we might presume that these responses are strongly suppressed in PNALT. In fact, immunosuppressive Treg are frequently present in HCV carriers, and the inhibitory activity has been demonstrated to be stronger in PNALT subjects than in patients with chronic active hepatitis [47]. Younger patients with CHC investigated in a previous study [21] and in the present study showed that the QQ homozygous variant is associated with a high HAI, a finding further supporting our hypothesis that the PNALT status may represent the terminal phase of an HCV chronic infection, when the active cellular immune response to infected hepatocytes present in CHC has burned itself out.

Also in other models of liver disease, the CB2-63 QQ variant was associated with low ALT serum values. In 438 Italian obese children with ultrasound-proven liver steatosis, the ALT serum values were lower in the 27 with the CB2-63 QQ variant (24.4±8 6IU/ml) than in the 222 with the QR variant (32.3±16 4IU/ml) and in the 189 with the RR variant (37.2±21 4IU/ml) [48]. Considering that the above-mentioned CB2-mediated inhibition of T-cell proliferation was normal in CB2-63 QQ subjects and reduced in RR subjects [6], [7], it has been hypothesized that the hepatic inflammation associated with liver steatosis is inhibited in CB2-63 QQ subjects [48], a hypothesis in good agreement with experimental observations showing the hepatoprotective properties of CB2 agonists in the mouse model of carbontetrachloride (CCl_4_)-induced liver injury. In fact, CCl_4_ induced acute hepatitis with higher serum ALT and AST levels and a more severe course in CB2 knock-out than in wild-type (WT) mice. In addition, ALT and AST levels were lower in CCl_4_-treated WT mice receiving the CB2 agonist JWH-133 [49].

Moreover, the stimulation of CB2 favors the transition of the Th1/Th2 balance to an anti-inflammatory Th2 profile by increasing the IL-10 levels [50]–[52]. In a study evaluating IL-10 administration in 30 patients with HCV-related liver disease, Nelson et al showed that IL-10 caused a decrease in the number of HCV-specific CD4+ and CD8+ IFN-gamma secreting T cells and alterations in PBMC cytokine production towards a Th2 dominant profile, as well as an improvement in the ALT serum levels [53].

PNALT subjects compared to patients in the abnormal ALT group showed a significantly lower BMI associated with a lower degree of liver steatosis, in good agreement with the recent demonstration that the enzyme that metabolizes the endocannabinoid 2-AG increases with the increase in the BMI, whereas the levels of the enzyme for the 2-AG synthesis do not change. Therefore, the endogenous levels of cannabinoids are higher in leaner subjects, and possibly exert a stronger CB2-mediated inhibition of the immune response [54].

Some limitations of the present study should also be underlined. Although interesting, the data on the role of the CB2-63 polymorphism on the ALT levels need confirmation in a multicenter prospective study on a larger cohort of patients. Further *in-vitro* studies are also needed to extend our knowledge on the CB2-63 QQ-related mechanisms inducing a suppression of liver cell necrosis. For a global understanding of the impact of the CB2-63 QQ variant on HCV chronic infection, some data are needed on patients with acute hepatitis C, liver cirrhosis with or without HCC and on orthotopic liver-transplanted patients. All 253 patients investigated were Italians born in southern Italy, but the present data need confirmation in more extensive studies including other ethnic populations. Although liver biopsy was obtained in a high prevalence (75%) of cases, a misclassification might have occurred in some of the non-biopsied cases. The present study, however, has clinical importance since it is the first study presenting significant data on the CB2-63 QQ variant as a newly identified genetic factor associated with different aspects of the clinical presentation of chronic HCV infection. In addition, the possible therapeutic implications of this new genetic factor cannot be excluded.

The data presented in this study encourage us to start a prospective multicenter studyand experimental studies on the role of the CB2-63 QQ variant in the different stages of HCV infection.
